# The Rationale of Complement Blockade of the MCP_ggaac_ Haplotype following Atypical Hemolytic Uremic Syndrome of Three Southeastern European Countries with a Literature Review

**DOI:** 10.3390/ijms241713041

**Published:** 2023-08-22

**Authors:** Daniel Turudic, Danka Pokrajac, Velibor Tasic, Dino Kasumovic, Zoltan Prohaszka, Danko Milosevic

**Affiliations:** 1 Department of Pediatrics, University Hospital Centre Zagreb, Kispaticeva 12, 10000 Zagreb, Croatia; 2 Pediatric Clinic, Clinical Center, University of Sarajevo, Patriotske Lige 81, 71000 Sarajevo, Bosnia and Herzegovina; dankapokrajac@hotmail.com; 3 Medical Faculty Skopje, University Children’s Hospital, 1010 Skopje, North Macedonia; vtasic2003@gmail.com; 4 Department of Nephrology and Dialysis, Dubrava University Hospital, School of Medicine, University of Zagreb, 10000 Zagreb, Croatia; dino.kasumovic9@gmail.com; 5 Department of Internal Medicine and Hematology, Semmelweis University, 1085 Budapest, Hungary; prohaszka.zoltan@med.semmelweis-univ.hu; 6 Research Group for Immunology and Haematology, Eotvos Lorand Research Network (Office for Supported Research Groups), Semmelweis University, 1085 Budapest, Hungary; 7 Croatian Academy of Medical Sciences, Kaptol ul. 15, 10000 Zagreb, Croatia; danko.milosevic@zg.t-com.hr; 8 Department of Pediatrics, Zabok General Hospital, and the Croatian Veterans Hospital, Bračak 8, 49210 Bračak, Croatia

**Keywords:** aHUS, complement blockade, MCPggaac, children, Southeastern Europe

## Abstract

We present eight cases of the homozygous MCPggaac haplotype, which is considered to increase the likelihood and severity of atypical hemolytic uremic syndrome (aHUS), especially in combination with additional risk aHUS mutations. Complement blockade (CBT) was applied at a median age of 92 months (IQR 36–252 months). The median number of relapses before CBT initiation (Eculizumab) was two. Relapses occurred within an average of 22.16 months (median 17.5, minimum 8 months, and maximum 48 months) from the first subsequent onset of the disease (6/8 patients). All cases were treated with PI/PEX, and rarely with renal replacement therapy (RRT). When complement blockade was applied, children had no further disease relapses. Children with MCPggaac haplotype with/without additional gene mutations can achieve remission through renal replacement therapy without an immediate need for complement blockade. If relapse of aHUS occurs soon after disease onset or relapses are repeated frequently, a permanent complement blockade is required. However, the duration of such a blockade remains uncertain. If complement inhibition is not applied within 4–5 relapses, proteinuria and chronic renal failure will eventually occur.

## 1. Introduction

The MCPggaac haplotype under additional complement-activating genetic conditions increases the probability and severity of atypical hemolytic uremic syndrome (aHUS) or at least activates secondary HUS [1,2,3,4,5,6]. This has been supported by the MCPggaac haplotype association with reduced gene transcription of membrane cofactor protein (MCP) in vitro [1,2,7,8,9]. Recently, it was found that MCPggaac haplotype carriers were at a significantly higher risk of graft loss and acute allograft rejection [3,10,11,12,13,14]. We analyzed follow-ups of all our patients with the MCPggaac haplotype in association with the occurrence and recurrence of aHUS. The cases were collected from three different countries: Croatia, Bosnia and Herzegovina, and North Macedonia. This study aimed to determine the prevalence of the MCPggaac haplotype in three Southeastern European countries, identify its adjacent mutations, and establish guidelines for complement blockade application in such cases.

## 2. Case Series

A total of fourteen children with genetically proven aHUS were enrolled in the study (six from Croatia, one from Bosnia and Herzegovina, and one from North Macedonia). All children were born to non-consanguine parents and had normal birth and growth history. They had no prior immune-mediated diseases. The male-to-female ratio was 50:50. We analyzed eight cases (of a total of fourteen) of homozygous MCPggaac haplotype with the combined additional risk of aHUS mutations. The MCPggaac haplotype was found to be the most prominent among aHUS mutations in regional populations.

The first onset of aHUS started at an average age of 44 months (median 33, IQR 24.5–66 months). The average follow-up time was 202 months (median 184, IQR 135.5–279 months). Turning 18 years old, two patients were transferred to adult care. The median age of the second relapse was 67.5 months (median 62, IQR 34–96 months). 

All patients had a sudden onset of pallor followed by well-known aHUS symptoms: fever, hemolytic anemia, thrombocytopenia, oliguria, erythrocyturia, and proteinuria. Our patients with MCPggaac haplotype or compound heterozygosity did not have any hypertensive crises [15]. Laboratory tests revealed normocytic anemia supported by ongoing hemolysis (low haptoglobin, elevated lactate dehydrogenase, aspartate transferase, and plasma-free hemoglobin). The results of immunohematological analyses (direct and indirect Coombs tests, anti-platelet/erythrocyte antibodies) were negative. Low C_3_ and normal C_4_ indicated an alternative pathway activation in all patients. Elevated factor H levels and C5b-9 terminal pathway activation markers were found in all cases. Normal complement C1q, factor B, and factor I, together with undetectable anti-C1q antibodies, supported pathological overactivation of the complement system and, in some cases, with overconsumption of complement factors. ADAMTS-13 levels were low but not deficient, which excluded TTP. The ISTH diagnostic scoring system for DIC guidelines was conducted and found to be negative. Common infective causes (O157:H7, Shigella sp., VTEC, Streptococcus pneumoniae) were excluded with negative stool, urine, throat and nasopharyngeal swabs, and other infective causes. Endomysial antibodies (EMA), ANCA, methylmalonic aciduria, hyperhomocysteinemia, and cobalamine deficiency were negative and were therefore excluded. All patients received prophylactic treatment with phenoxymethylpenicillin V as well as vaccination against meningococcal groups A, C, W-135, and Y and meningococcal group B prior to complement blockade therapy.

**Figure 1:**
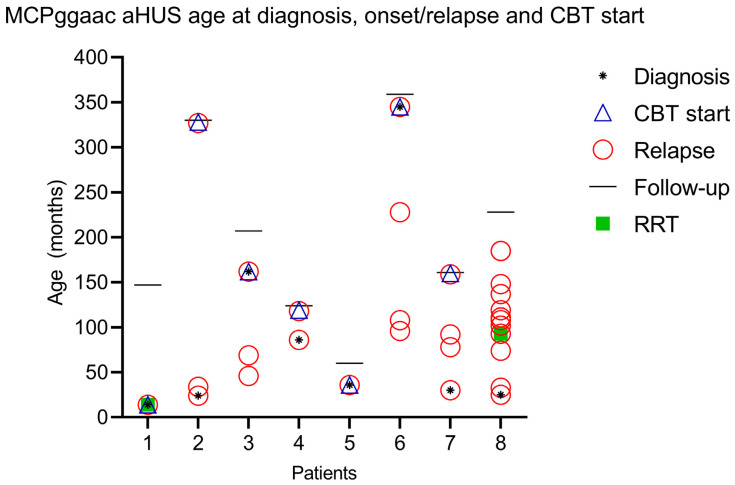
Graphical representation of age at diagnosis, onset, and start of complement blockade therapy (CBT).

Complement blockade was applied at the age median of 92 months (IQR 36–252 months) with an average number of relapses before complement blockade with eculizumab after two episodes. In case 1, a complement blockade was implemented after two weeks of intensive care unit treatment after prolonged hemolysis with complement overamplification and overconsumption. Two children were siblings (case 2 and case 3). In case 2, an older sister, an expectant attitude was adopted after two relapses. Complement blockade was implemented after the third relapse of the disease with a follow-up of 327 months when she was an adult (follow-up of 303 months, 25.25 years). In a genetically similar case (younger brother, case 3), complement blockade was applied after two relapses. In case 4, despite positive genetic MCPggaac haplotype analysis having been made after the first onset of aHUS, complement blockade was implemented after the second relapse, after a hemolysis-free period of 116 months. Case 5 had acute lymphoblastic leukemia prior to aHUS onset and was treated according to the BFM ALL IC-2009 protocol. She achieved early remission with negative minimal residual disease (MRD) on day 33 and <1% blasts in bone marrow aspirate. There were no signs of leukemia relapse at the time of aHUS diagnosis. In case 6, we applied complement blockade after the fourth relapse when a proper diagnosis was made at adult age, at which a low range of proteinuria persists permanently without altering the global renal function. The child of case 7 had MCPggaac heterozygous mutation alongside three additional different CHF gene mutations, and we applied complement blockade after the fourth relapse of aHUS. In case 8, after ten relapses, complement blockade was not administered due to the inaccessibility of such therapy, with the onset of proteinuria detected after the fifth relapse and permanent renal insufficiency after the seventh relapse (Figure 1). 

All patients received PI/PEX (8/8), and only two patients received renal replacement therapy (RRT) (case 1, case 8). Case 1 received RRT at the onset of the disease, and case 8 at the fourth relapse of the disease. RRT was applied in case 8 after each subsequent relapse (Figure 1). The patient now has permanent renal deterioration and is currently awaiting renal transplantation. 

Most of the relapses prior to complement blockade occurred within an average of 22.16 months (median 17.5, minimum 8 months, and 48 months maximum) since the first next onset of the disease (6/8 patients).

All cases with applied complement blockade had no new relapses of the disease, with the longest follow-up of 123 months. One child stopped receiving complement blockade after 11 months (case 5) with no relapse after 60 months of follow-up, and an adult patient (case 7) stopped receiving complement blockade after only 6 months of treatment with no relapse after 14 months of follow-up. 

The additional mutations in the aHUS spectrum are mainly heterozygous complement factor H mutations (CHF spectrum, cases 1, 2, 4, 7, 8), CD 46 (cases 2, 3, 4, 6, 8), C3 (cases 1,4), and one homozygous CFH H3 mutation (case 5) (Appendix A).

Genetic analysis was performed by multiplex ligation-dependent probe amplification (MLPA) to reveal deletions or duplications in CFH, CFHR-1, -2, -3, -4, and -5 genes. The DNA sequence of the whole coding regions of the complement factor H gene (CFH, exon 1–9, 11–23), complement factor I gene (CFI, exon 1–13), membrane cofactor protein gene (CD46, exon 1–14), complement C3 gene (C3, exon 1–41), complement factor B gene (CFB, exons 1–18), thrombomodulin gene (THBD, exon 1), and complement factor H-related protein 5 gene (CFHR5, exons 1–10) was determined by direct DNA sequencing of polymerase chain reaction (PCR) products amplified from the total genomic DNA.

A comprehensive literature search was performed to assess the incidence and outcomes of aHUS patients with the homozygous MCPggaac haplotype. Four publicly available databases were searched: Medline via PubMed, Scopus, Web of Science Core Collection, and Google Scholar. We used the search term “MCPggaac” for all searches. The search strategy included keywords, MeSH terms, and any text word to maximize the literature output. The search engine was last accessed on 10 June 2023, and all available full-text articles until July 2023 were included. No time limits were set. No search filters or limits were used, and all articles were included. Language barriers were non-existent. Investigators independently reviewed titles, abstracts, and full-text articles. Disagreements regarding study inclusion were resolved by consensus of the investigators. Both pediatric and adult populations were included. Only studies of the MCPggaac haplotype in vivo were included. Studies on cell culture or animal experiments are considered in the Discussion section. The database searches resulted in 20 articles in total. The deduplication process was performed with EndNote ver. 20. Four additional articles were added following the citation search. Microsoft Excel was used to organize raw data after its extraction (Table 1). We concentrated mainly on articles dealing with the homozygous haplotype and articles dealing with the heterozygous form as needed.

## 3. Discussion

Articles addressing follow-up of the MCPggaac haplotype in aHUS patients are still scarce. The MCPggaac haplotype comprises two SNPs in the promoter region and has been associated with a two- to three-fold increased risk of aHUS [2,9,16,17,20,30,31]. The aHUS-associated MCPggaac haplotype extends over a large portion of the RCA gene cluster, including the C4BP, DAF, CR1, and MCP genes [2]. This can encompass c.−652A>G (rs2796267), c.−366A>G (rs2796268), c.IVS9−78G>A (rs1962149), c.IVS12+638G>A (rs859705), and c.4070T>C (rs7144) polymorphisms. The MCPggaac haplotype has been associated with aHUS in sporadic and familial cases [9,17,32]. It seems that the clinical expression of the homozygous MCPggaac haplotype depends significantly on additional mutations in the aHUS spectrum, especially CFH and CFI, and if so, various infectious diseases or even drugs trigger aHUS in children who are genetically prone to aHUS onset [1,2,8,9,29,33,34,35,36,37]. MCPggaac compound heterozygosity with additional risk polymorphisms can lead to repeated aHUS relapses and renal deterioration [18]. Even though the MCPggaac haplotype might not have an additional effect on MCP expression in all cases, the MCPggaac haplotype acts as a strong risk variant of aHUS onset or serves as a compound heterozygosity added to other heterozygous mutations (case 7). MCPggaac polymorphism is associated with a reduced risk of relapse and late aHUS onset in the absence of trigger and/or additional aHUS mutations [27].

The concurrence of different complement regulatory gene mutations and polymorphisms (CFH, MCP, or IF) increases the predisposition for aHUS development. It is theorized that the MCPggaac haplotype may have an additive effect in further reducing the expression of MCP in carriers of the MCP mutation [2]. This view was recently discussed in an article on the expression of MCP on granulocytes and endothelial cells, which found no difference between the wild-type and the MCPggaac haplotype [38]. The amount of literature data is insufficient to suggest that the homozygous MCPggaac haplotype could be sufficient for aHUS onset because, in most cases, there are also other homozygous or numerous heterozygous mutations.

The MCPggaac homozygous haplotype is prevalent among our aHUS mutations, and such a haplotype with additional heterozygous aHUS mutations should be expected in our population. Unaffected persons can carry one or two genetic risk factors which suggest that a combination of mutations and the risk haplotype are critical in aHUS development [22,32]. Indeed, the MCPggaac haplotype is associated with lower levels of this receptor on the cell surface and, if linked with the CHF-H3 risk haplotype with a lower plasma level of CHF, is prone to recidivate and manifests as a more severe onset of aHUS [1,9,21,31,36,37,39,40]. In our case series, we conform to such an opinion as most of our cases comprise at least one or several other mutations (CHF, CD 46, and C3), which probably act as compounding to aHUS onset. However, how much MCPggaac additional mutations contribute to total compound heterozygosity and aHUS onset risk remains open. In long-term follow-up, one patient of the MCPggaac haplotype with homozygous CH46 and heterozygous CHF mutations is now in end-stage renal failure (case 8), as was already described previously by other authors [17]. Contrarily, one child with MCPggaac haplotype and homozygous CHF H3 mutation, despite the withdrawal of complement blockade, remained disease-free for a considerable time period.

The discovery of complement blockade fundamentally changed the treatment approach to aHUS. A humanized monoclonal antibody binds to complement protein C5, thereby blocking its cleavage. Therefore, the production of the complement terminal component C5a as well as the membrane attack complex (MAC) C5b-9 is prevented. The use of complement blockade (especially the newer versions) is safe and effective in children and adults [41,42]. Complement blockade should be administered immediately in life-threatening conditions in case of signs of complement overactivation and overconsumption. Immediate complement blockade should be performed in patients with unequivocal clinical and laboratory signs of aHUS. In doubtful cases, when the child is in a favorable overall condition, with maintained renal function, GFR and diuresis, and C3 within reference values, with rapid clinical improvement on PI/PEX treatment, it seems acceptable to wait for genetic analysis [25,32,43]. Then, with such a decision, the sentences of *conditio sine qua non* and *primum non nocere* should have adhered. Actually, a patient (case 7) was in the full remission phase with only PI/PEX administration until genetic analysis arrived. The new approach emphasizes cost-effectiveness and rational cost reduction, which is to be encouraged. Nevertheless, such a clinical decision on cost-effectiveness reasoning should be taken with caution [44]. Signs of overactivation and overconsumption should be carefully monitored to ensure timely administration of complement blockade. It would be beneficial to monitor the parameters of hemolysis (bilirubin, AST, LDH, platelet, reticulocytes, proteinuria, and haptoglobin) and preferably the C5-b9 level in order to intervene in time with complement blockade. Without apparent alternative complement pathway activation, an early application of complement blockade cannot be rationally supported [24]. Actually, an adult aHUS patient with the MCP gene (case 8) with c.287–2A > G (splice acceptor) mutation and compound heterozygosity for CFH-H3 and MCPggaac haplotype, who had an initially infection-triggered mild disease course, was treated with PI/PEX and RRT only after evidence of renal deterioration [44]. The onset of the aHUS is mostly triggered by mostly unknown infective causes save the 6-mercaptopurine maintenance therapy for acute leukemia (case 5). This particular child was considered for immediate complement blockade to avoid a relapse of a serious underlying disease (acute lymphoblastic leukemia) [45]. In rare cases of long-term life-threatening hemolysis with multiorgan failure, blockade of complement has shown a beneficial effect even in patients whose genetic background has not been unequivocally proven [3,28,46,47,48].

Some of our cases were not given complement blockade therapy for various reasons. One sibling was denied complement blockade despite her brother receiving similar mutations. The reason behind such a decision was her brother’s younger age and because of the absence of overamplification and overconsumption of complement during aHUS onset (cases 2 and 3). A long distance from the first two onsets, with more than two decades after the last onset of aHUS, seems to prove the plausibility for such a decision. However, a complement blockade was applied in her adult age after the third aHUS relapse to avoid further kidney damage (case 2). Within a similar time expectancy, one patient was treated with PI/PEX (plasma infusion/plasma exchange) alone and has been disease-free for 5 years (case 4). After such a prolonged disease-free period, a complement blockade was applied after the second aHUS onset. This patient was clearly misjudged as being an aHUS patient and was therefore not genetically tested, thus not receiving complement blockade until adult age (case 7). Recently, she received complement blockade after the fourth relapse of hemolysis, when an MCPggaac haplotype was determined. Despite complement blockade, proteinuria remained permanent.

If relapse of aHUS occurs soon after disease onset or relapses are repeated frequently, permanent complement blockade is required, but the duration of such a blockade remains uncertain. However, according to their additional genetic background in combination with the MCPggaac haplotype, some patients need immediate complement blockade [49,50]. Contrarily, the PI/PEX success in ceasing the hemolysis may lead to the misjudgment of having typical HUS, thus neglecting the genetic analysis.

Removal of complement blockade in the case of the MCPggaac haplotype is doubtful as this haplotype increases the relapse risk, penetrance, and disease severity of aHUS [1,16,18,26]. Younger age (toddlers), life-threatening disease, permanent renal damage, or active aHUS that does not return to normal values or reduced GFR necessitate permanent complement blockade [51]. Recent articles recommend withdrawal of the complement blockade after three months of treatment with the remark that a definitive opinion requires larger data registries [43]. However, in the case of living renal kidney transplantation, a longer prophylactic period should be considered [10,52]. It is recommended to control the plasma level of C5b-9 before the decision to withdraw complement blockade to prevent relapses of aHUS [53,54]. If the decision of complement blockade cessation is made, it seems plausible to carefully monitor clinical and laboratory signs of kidney damage, as was afore recommended (especially in case of infection-triggered aHUS [38,50].) Our results indicate that if aHUS relapse occurs quickly after the onset of the disease or relapses are repeated frequently, permanent blockade of complement is necessary to avoid further kidney damage. If complement inhibition is not applied within 4–5 relapses of MCPggac-generated aHUS, proteinuria and chronic renal failure will eventually occur.

We believe that the MCPggaac haplotype with additional gene mutations or MCPggaac compound heterozygosity should be treated with complement blockade in three cases: frequent relapses with renal damage and/or rapid return of the disease after the abolition of complement blockade, or in case of serious permanent damage of organs (kidney, CNS, etc.).

## 4. Conclusions

Although the MCPggaac haplotype with the onset and relapse of additional gene mutations can achieve remission by PI/PEX without complement inhibition, the disease often relapses quickly. While in aHUS, patients following homozygous genetic mutations (most notably complement factor H, CHF) should be given a proper complement blockade as soon as possible, an MCPggaac haplotype compounding with other heterozygous mutations (most notably CHF) is prone to recidivate episodes of aHUS. In undisputed cases, immediate complement should be applied, while in doubtful cases, it should be avoided as much as possible. With aHUS onset and underlying diseases (most notably hematologic ones) existing, a complement blockade should be implemented immediately to prevent the activation of underlying disease. If complement inhibition is not applied within 4–5 relapses after MCPggaac haplotype onset, with/without adjacent mutations (CHF, CD46, C3), proteinuria, renal damage, and eventually chronic renal failure will occur.

## Figures and Tables

**Table 1:** MCPggaac homozygous polymorphisms described in the literature.

Article	Population	Year	Gene: Variant or Haplotype	Risk Genotype	Additional Genotypes
Fang et al. [16]		2008	*MCP: MCPggaac*	homozygous	*heterozygous R69W MCP and N151S CFI mutations, heterogzygous CFI and c. 905-925del21n mutations*
Lhotta [17]		2009	*MCP: MCPggaac*	homozygous	*het C3 R570Q mutation,*
Obando et al. [18]	Spain	2012	*MCP: MCPggaac*	homozygous	
Pelicano et al. [19]	Spain	2013	*MCP:MCPggaac*	homozygous	*het CFHcataag (protective haplotype)*
Szarvas et al. [20]		2014	*MCP: MCPggaac and CFH H3*	homozygous, heterozygous, compound heterozgyous	*Het CFH Y402H; hom CFH Y402H; het CFH E936D; het C3 R102G; het C3 P314L; het CFB R32W; het CFB L9H; het CFH V62I; het CFB R32Q;*
Martínez-Barricarte et al. [21]	Spain	2015	*MCP: MCPggaac*	homozygous	
			*MCP: MCPggaac*	heterozygous	*CFH_GATAAG_*
			*MCP: MCPggaac*	heterozygous	*CFH_GATAAG_*
			*MCP: MCPggaac*	heterozygous	*CFH_TGTGGT_ (homozygous)*
			*MCP: MCPggaac*	heterozygous	*CFH_TGTGGT_ (homozygous)*
			*MCP: MCPggaac*	heterozygous	*CFH_CATAAG_ (heterozygous)*
Valoti et al. [22]	Italy	2015	MCPallele c.*897 T.C (rs7144)	homozygous	*het CFH-H3 (TGTGT)*
Monteavaro et al. [23]	Spain	2016	*MCP: MCPggaac*	homozygous	*CFH/CFHR1 hybrid gene, heterozygous CFH (H3)*
Fidalgo et al. [4]	Spain/Portugal	2017	*CFH H3:MCPggaac*	compound heterozygous	
Marini et al. [24]	Portugal	2019	*MCP: MCPggaac*	het (compound)	*MCP c.287–2A > G (splice acceptor), MCP_ggaac_ and CFH-H3 (compound heterozygous)*
Flögelová et al. [25]	Czechia	2020	*MCPggaac haplotype of CD46 gene*	heterogygous	*MCP (CD46) p.C35Y (heterozygous)*
Le Clech et al. [3]		2020	*MCPggaac haplotype of CD46 gene*	homozygous	
Levart et al. [26]	Slovenia	2020	*MCP: MCPggaac*	homozygous	*heterozygous variation (H508H), heterozygous CHF V621 missense variation*
Lumbreras et al. [27]	Spain	2020	*MCP: MCPggaac*	homozygous, heterogyzous	MCP: Gly243Val, CFI: Gly162Asp, CFH:Arg885Serfs*13, *THBD: (Ala43Thr)*
Timmermans et al. [15]		2020	*MCP: MCPggaac*	homozygous, heterogyzous	*C3 c.463A > C* *
Petr V et al. ** [10]	Czechia	2022	*MCPggaac c.-652A/G, rs2796267*	heterogygous	*CFH, CD46, C3, CFB*
	Czechia	2022	*MCPggaac c.-366A/G, rs2796268*	heterogygous	*CFH, CD46, C3, CFB*
	Czechia	2022	*MCPggaac IVS9-78G/A (c.989-78G > A), rs1962149*	heterogygous	*CFH, CD46, C3, CFB*
Rysava et al. [28]	Czechia	2022	*MCPggaac haplotype of CD46 gene*	heterogygous	*CFH (c.3096C > A, p.C1032X) (heterozygous)*
Van. Herpt et al. [8]	Netherlands	2022	*MCPggaac*	heterogygous	*C2 c.841_849+19del, deletion of CFHR1 and CFHR3 (all heterozygous)*
Jelicic I. et al. [29]	Croatia	2023	*MCPggaac*	homozygous	*Heterozygous CD46 gene (c.286+2 T > G) splice site mutation, rare heterozygous variant (c.463A > C), homozygous for the CFH H3 haplotype (with the rare alleles c.-331C > T, Q672Q and E936D polymorphisms)*

* The risk haplotype MCPggaac is formed by rs2796267, rs2796268, rs1962149, rs859705, and rs7144. ** The study also involves rs859705 (IVS12638A/G) and rs7144 (c.2232C/T) which are strongly linked to the IVS9−78G/A variation.

## Data Availability

The raw data supporting the conclusions of this article will be made available by the authors, without undue reservation.

## Appendix A

**Table A1:** Additional (besides MCPggaac haplotype) variants of patients with aHUS.

Patient	CD46, MCPggaac Risk Haplotype	Additional Variants
Patient 1	homozygous MCPggaac	heterozygous *C3* E1160K (LPV) heterozygous *CFH* H3 haplotype (risk haplotype *)
Patient 2	homozygous MCPggaac	heterozygous *CD46* S274I gene (VUS) heterozygous *CFH* H3 haplotype (risk haplotype *)
Patient 3	homozygous MCPggaac	heterozygous *CD46* S274I gene (VUS)
Patient 4	homozygous MCPggaac	heterozygous *CD46* c.286 + 2T > G (PV) heterozygous *C3* gene N1229N (LBV) heterozygous *CFH* c.-331C > T (risk variant)
Patient 5	homozygous MCPggaac	homozygous *CFH* H3 (risk haplotype *)
Patient 6	homozygous MCPggaac	homozygous *CD46* c.286 + 2T > G (PV) heterozygous *CFB* Y67H (VUS)
Patient 7	heterozygous MCPggaac	heterozygous *CFHR5* K144N (LBV) heterozygous *CFH* Q672Q and E936D (risk variants) heterozygous *CFH* V62I (protective variant)
Patient 8	homozygous MCPggaac	homozygous *CD46* c.286 + 2T > G (PV) heterozygous *CFH* c.-331C > T (risk variant)

* The risk haplotype MCPggaac is formed by rs2796267, rs2796268, rs1962149, rs859705, and rs7144.

The presence of a previously reported risk haplotype is predicted based on the genotype of the polymorphisms of the corresponding gene.

Legend: Interpretative categories of variants identified in aHUS patients.

PV	Pathogenic variant
LPV	Likely pathogenic variant
VUS	A variant of uncertain significance
LBV	Likely benign variant
BV	Benign variant

## References

[1] Esparza-Gordillo J., Jorge E., Garrido C.A., Carreras L., López-Trascasa M., Sánchez-Corral P., Decordoba S. (2006). Insights into hemolytic uremic syndrome: Segregation of three independent predisposition factors in a large, multiple affected pedigree. Mol. Immunol..

[2] Esparza-Gordillo J., Goicoechea de Jorge E., Buil A., Carreras Berges L., López-Trascasa M., Sánchez-Corral P., de Córdoba S.R. (2005). Predisposition to atypical hemolytic uremic syndrome involves the concurrence of different susceptibility alleles in the regulators of complement activation gene cluster in 1q32. Hum. Mol. Genet..

[3] Le Clech A., Simon-Tillaux N., Provôt F., Delmas Y., Vieira-Martins P., Limou S., Halimi J.-M., Le Quintrec M., Lebourg L., Grangé S. (2019). Atypical and secondary hemolytic uremic syndromes have a distinct presentation and no common genetic risk factors. Kidney Int..

[4] Fidalgo T., Martinho P., Pinto C.S., Oliveira A.C., Salvado R., Borràs N., Coucelo M., Manco L., Maia T., Mendes M.J. (2017). Combined study of ADAMTS13 and complement genes in the diagnosis of thrombotic microangiopathies using next-generation sequencing. Res. Pract. Thromb. Haemost..

[5] Praga M., Rodríguez de Córdoba S. (2019). Secondary atypical hemolytic uremic syndromes in the era of complement blockade. Kidney Int..

[6] Bernabéu-Herrero M.E., Jiménez-Alcázar M., Anter J., Pinto S., Sánchez Chinchilla D., Garrido S., López-Trascasa M., de Córdoba S.R., Sánchez-Corral P. (2015). Complement factor H, FHR-3 and FHR-1 variants associate in an extended haplotype conferring increased risk of atypical hemolytic uremic syndrome. Mol. Immunol..

[7] Wu X., Liszewski M., Java A., Atkinson J. (2023). Atypical Hemolytic Uremic Syndrome: Genetically-Based Insights into Pathogenesis through an Analysis of the Complement Regulator CD46. Annals of Blood. https://aob.amegroups.org/article/view/7737.

[8] van Herpt T.T.W., Timmermans S.A.M.E.G., van Mook W.N.K.A., van Bussel B.C.T., van der Horst I.C.C., Maessen J.G., Natour E., van Paassen P., Heuts S. (2022). Postsurgical Thrombotic Microangiopathy and Deregulated Complement. J. Clin. Med..

[9] Arjona E., Huerta A., de Jorge E.G., de Córdoba S.R. (2020). Familial risk of developing atypical hemolytic-uremic syndrome. Blood.

[10] Petr V., Csuka D., Hruba P., Szilágyi Á., Kollar M., Slavcev A., Prohászka Z., Viklicky O. (2022). MCPggaac haplotype is associated with poor graft survival in kidney transplant recipients with de novo thrombotic microangiopathy. Front. Immunol..

[11] Park M., Kim S., Lee T., Lee S., Moon J., Ihm C., Kim Y., Kang S., Jeong K., Chung J.-H. (2016). A Promoter Polymorphism in the CD46 Complement Regulatory Protein Gene Is Associated with Acute Renal Allograft Rejection. Transplant. Proc..

[12] Verhave J.C., Westra D., van Hamersvelt H.W., van Helden M., Kar N.C., Wetzels J.F. (2013). Living kidney transplantation in adult patients with atypical haemolytic uraemic syndrome. Neth. J. Med..

[13] Sánchez-Moreno A., Cerda F., Rodríguez-Barba A., Fijo J., Bedoya R., Arjona E., de Córdoba S.R. (2020). Is the atypical hemolytic uremic syndrome risk polymorphism in Membrane Cofactor Protein *MCPggaac* relevant in kidney transplantation? A case report. Pediatr. Transplant..

[14] Rodríguez de Córdoba S. (2022). Genetic variability shapes the alternative pathway complement activity and predisposition to complement-related diseases. Immunol. Rev..

[15] Timmermans S.A., Wérion A., Damoiseaux J.G., Morelle J., Reutelingsperger C.P., van Paassen P. (2020). Diagnostic and Risk Factors for Complement Defects in Hypertensive Emergency and Thrombotic Microangiopathy. Hypertension.

[16] Fang C.J., Fremeaux-Bacchi V., Liszewski M.K., Pianetti G., Noris M., Goodship T.H.J., Atkinson J.P. (2008). Membrane cofactor protein mutations in atypical hemolytic uremic syndrome (aHUS), fatal Stx-HUS, C3 glomerulonephritis, and the HELLP syndrome. Blood.

[17] Lhotta K., Janecke A.R., Scheiring J., Petzlberger B., Giner T., Fally V., Würzner R., Zimmerhackl L.B., Mayer G., Fremeaux-Bacchi V. (2009). A Large Family with a Gain-of-Function Mutation of Complement C3 Predisposing to Atypical Hemolytic Uremic Syndrome, Microhematuria, Hypertension and Chronic Renal Failure. Clin. J. Am. Soc. Nephrol..

[18] Obando I., Camacho M.S., Falcon-Neyra D., Hurtado-Mingo A., Neth O. (2012). Atypical Hemolytic Uremic Syndrome Associated with Bordetella pertussis Infection. Pediatr. Infect. Dis. J..

[19] Pelicano M.B., de Córdoba S.R., Diekmann F., Saiz M., Herrero S., Oppenheimer F., Campistol J.M. (2013). Anti-C5 as Prophylactic Therapy in Atypical Hemolytic Uremic Syndrome in Living-Related Kidney Transplantation. Transplantation.

[20] Szarvas N., Szilágyi Á., Tasic V., Nushi-Stavileci V., Sofijanova A., Gucev Z., Szabó M., Szabó A., Szeifert L., Reusz G. (2014). First-line therapy in atypical hemolytic uremic syndrome: Consideration on infants with a poor prognosis. Ital. J. Pediatr..

[21] Martínez-Barricarte R., Heurich M., López-Perrote A., Tortajada A., Pinto S., López-Trascasa M., Sánchez-Corral P., Morgan B.P., Llorca O., Harris C.L. (2015). The molecular and structural bases for the association of complement C3 mutations with atypical hemolytic uremic syndrome. Mol. Immunol..

[22] Valoti E., Alberti M., Tortajada A., Garcia-Fernandez J., Gastoldi S., Besso L., Bresin E., Remuzzi G., Rodríguez de Cordoba S., Noris M. (2015). A Novel Atypical Hemolytic Uremic Syndrome–Associated Hybrid CFHR1/CFH Gene Encoding a Fusion Protein That Antagonizes Factor H–Dependent Complement Regulation. J. Am. Soc. Nephrol..

[23] García Monteavaro C., Peralta Roselló C., Quiroga B., Baltar Martín J.M., Castillo Eraso Eraso L., de Álvaro Moreno F., Martínez Vea A., Visus-Fernández de Manzanos M.T. (2016). Adjustment of Eculizumab Dosage Pattern in Patients with Atypical Hemolytic Uremic Syndrome with Suboptimal Response to Standard Treatment Pattern. Case Rep. Nephrol..

[24] Matošević M., Kos I., Davidović M., Ban M., Matković H., Jakopčić I., Brinar I.V., Szilágyi Á., Csuka D., Sinkovits G. (2023). Hemolytic uremic syndrome in the setting of COVID-19 successfully treated with complement inhibition therapy: An instructive case report of a previously healthy toddler and review of literature. Front. Pediatr..

[25] Flögelová H., Volejníková J., Hrachovinová I., Prohászka Z., Šeda M., Gumulec J. (2020). Repeated spontaneous remission of atypical hemolytic-uremic syndrome caused by influenza—A case report. [Opakovaná spontánní remise atypického hemolyticko-uremického syndromu vyvolaného chřipkou—Kazuistika]. Czecho-Slovak Pediatr./Cesko-Slov. Pediatr..

[26] Fakhouri F., Fila M., Hummel A., Ribes D., Sellier-Leclerc A.-L., Ville S., Pouteil-Noble C., Coindre J.-P., Le Quintrec M., Rondeau E. (2021). Eculizumab discontinuation in children and adults with atypical hemolytic-uremic syndrome: A prospective multicenter study. Blood.

[27] Lumbreras J., Subias M., Espinosa N., Ferrer J.M., Arjona E., Rodríguez de Córdoba S. (2020). The Relevance of the MCP Risk Polymorphism to the Outcome of aHUS Associated with C3 Mutations. A Case Report. Front. Immunol..

[28] Galic S., Csuka D., Prohászka Z., Turudic D., Dzepina P., Milosevic D. (2019). A case report of a child with sepsis induced multiorgan failure and massive complement consumption treated with a short course of Eculizumab: A case of crosstalk between coagulation and complement?. Medicine.

[29] Jelicic I., Kovacic V., Luketin M., Mikacic M., Skaro D.B. (2023). Atypical HUS with multiple complement system mutations triggered by synthetic psychoactive drug abuse: A case report. J Nephrol..

[30] Kavanagh D., Goodship T.H., Richards A. (2013). Atypical Hemolytic Uremic Syndrome. Semin. Nephrol..

[31] Bresin E., Rurali E., Caprioli J., Sanchez-Corral P., Fremeaux-Bacchi V., Rodríguez de Cordoba S., Pinto S., Goodship T.H., Alberti M., Ribes D. (2013). Combined Complement Gene Mutations in Atypical Hemolytic Uremic Syndrome Influence Clinical Phenotype. J. Am. Soc. Nephrol..

[32] Bu F., Borsa N., Gianluigi A., Smith R.J.H. (2012). Familial Atypical Hemolytic Uremic Syndrome: A Review of Its Genetic and Clinical Aspects. Clin. Dev. Immunol..

[33] Fremeaux-Bacchi V., Fakhouri F., Garnier A., Bienaimé F., Dragon-Durey M.-A., Ngo S., Moulin B., Servais A., Provot F., Rostaing L. (2013). Genetics and Outcome of Atypical Hemolytic Uremic Syndrome: A nationwide French series comparing children and adults. Clin. J. Am. Soc. Nephrol..

[34] Fremeaux-Bacchi V., Kemp E.J., Goodship J.A., Dragon-Durey M.-A., Strain L., Loirat C., Deng H.-W., Goodship T.H.J. (2005). The development of atypical haemolytic-uraemic syndrome is influenced by susceptibility factors in factor H and membrane cofactor protein: Evidence from two independent cohorts. J. Med. Genet..

[35] Manenti L., Gnappi E., Vaglio A., Allegri L., Noris M., Bresin E., Pilato F.P., Valoti E., Pasquali S., Buzio C. (2013). Atypical haemolytic uraemic syndrome with underlying glomerulopathies. A case series and a review of the literature. Nephrol. Dial. Transplant..

[36] Caprioli J., Castelletti F., Bucchioni S., Bettinaglio P., Bresin E., Pianetti G., Gamba S., Brioschi S., Daina E., Remuzzi G. (2003). Complement factor H mutations and gene polymorphisms in haemolytic uraemic syndrome: The C-257T, the A2089G and the G2881T polymorphisms are strongly associated with the disease. Hum. Mol. Genet..

[37] Goicoechea de Jorge E., Harris C.L., Esparza-Gordillo J., Carreras L., Arranz E.A., Garrido C.A., López-Trascasa M., Sánchez-Corral P., Morgan B.P., Rodríguez de Córdoba S. (2007). Gain-of-function mutations in complement factor B are associated with atypical hemolytic uremic syndrome. Proc. Natl. Acad. Sci. USA.

[38] Frimat M., Roumenina L.T., Tabarin F., Halbwachs-Mecarelli L., Fremeaux-Bacchi V. (2012). Membrane cofactor protein (MCP) haplotype, which predisposes to atypical hemolytic and uremic syndrome, has no consequence on neutrophils and endothelial cells MCP levels or on HUVECs ability to activate complement. Immunobiology.

[39] Caprioli J., Noris M., Brioschi S., Pianetti G., Castelletti F., Bettinaglio P., Mele C., Bresin E., Cassis L., Gamba S. (2006). Genetics of HUS: The impact of MCP, CFH, and IF mutations on clinical presentation, response to treatment, and outcome. Blood.

[40] Provaznikova D., Rittich S., Malina M., Seeman T., Marinov I., Riedl M., Hrachovinova I. (2011). Manifestation of atypical hemolytic uremic syndrome caused by novel mutations in MCP. Pediatr. Nephrol..

[41] Rondeau E., Scully M., Ariceta G., Barbour T., Cataland S., Heyne N., Miyakawa Y., Ortiz S., Swenson E., Vallee M. (2020). The long-acting C5 inhibitor, Ravulizumab, is effective and safe in adult patients with atypical hemolytic uremic syndrome naïve to complement inhibitor treatment. Kidney Int..

[42] Ariceta G., Dixon B.P., Kim S.H., Kapur G., Mauch T., Ortiz S., Vallee M., Denker A.E., Kang H.G., Greenbaum L.A. (2020). The long-acting C5 inhibitor, ravulizumab, is effective and safe in pediatric patients with atypical hemolytic uremic syndrome naïve to complement inhibitor treatment. Kidney Int..

[43] Bouwmeester R.N., Duineveld C., Wijnsma K.L., Bemelman F.J., van der Heijden J.W., van Wijk J.A., Bouts A.H., van de Wetering J., Dorresteijn E., Berger S.P. (2022). Early Eculizumab Withdrawal in Patients with Atypical Hemolytic Uremic Syndrome in Native Kidneys Is Safe and Cost-Effective: Results of the CUREiHUS Study. Kidney Int. Rep..

[44] Marini S.C., Gomes M., Guilherme R., Carda J.P., Pinto C.S., Fidalgo T., Ribeiro M.L. (2019). Atypical hemolytic–uremic syndrome: Recurrent phenotypic expression of a patient with MCP gene mutation combined with risk haplotypes. Blood Coagul. Fibrinolysis.

[45] Turudic D., Milosevic D., Bilic K., Prohászka Z., Bilic E. (2022). A Limited Course of Eculizumab in a Child with the Atypical He-molytic Uremic Syndrome and Pre-B Acute Lymphoblastic Leukemia on Maintenance Therapy Case Report and Literature Review. J. Clin. Med..

[46] Alizadeh F., O’halloran A., Alghamdi A., Chen C., Trissal M., Traum A., DeCourcey D. (2021). Toddler with New Onset Diabetes and Atypical Hemolytic-Uremic Syndrome in the Setting of COVID-19. Pediatrics.

[47] Mahajan R., Lipton M., Broglie L., Jain N.G., Uy N.S. (2020). Eculizumab treatment for renal failure in a pediatric patient with COVID-19. J. Nephrol..

[48] Aurora T., Joseph N., Bhoopalan S.V., Caniza M.A., Flerlage T., Ghafoor S., Hankins J., Hijano D.R., Jesudas R., Kirkham J. (2022). The successful use of eculizumab for treatment of thrombotic microangiopathy in pediatric acute SARS-CoV-2 infection and multisystem inflammatory syndrome in children. Haematologica.

[49] Rysava R., Peiskerova M., Tesar V., Benes J., Kment M., Szilágyi Á., Csuka D., Prohászka Z. (2022). Atypical hemolytic uremic syndrome triggered by mRNA vaccination against SARS-CoV-2: Case report. Front. Immunol..

[50] Walle J.V., Delmas Y., Ardissino G., Wang J., Kincaid J.F., Haller H. (2017). Improved renal recovery in patients with atypical hemolytic uremic syndrome following rapid initiation of eculizumab treatment. J. Nephrol..

[51] Levart T.K. (2020). A child with atypical hemolytic uremic syndrome: Is there a rationale to stop eculizumab?. Clin. Nephrol..

[52] Timmermans S.A., Damoiseaux J.G., Werion A., Reutelingsperger C.P., Morelle J., van Paassen P. (2021). Functional and Genetic Landscape of Complement Dysregulation Along the Spectrum of Thrombotic Microangiopathy and its Potential Implications on Clinical Outcomes. Kidney Int. Rep..

[53] Fakhouri F., Fila M., Provôt F., Delmas Y., Barbet C., Châtelet V., Rafat C., Cailliez M., Hogan J., Servais A. (2016). Pathogenic Variants in Complement Genes and Risk of Atypical Hemolytic Uremic Syndrome Relapse after Eculizumab Discontinuation. Clin. J. Am. Soc. Nephrol..

[54] Cugno M., Gualtierotti R., Possenti I., Testa S., Tel F., Griffini S., Grovetti E., Tedeschi S., Salardi S., Cresseri D. (2014). Complement functional tests for monitoring eculizumab treatment in patients with atypical hemolytic uremic syndrome. J. Thromb. Haemost..

